# Clinical Efficacy and Safety of Pamidronate Therapy on Bone Mass Density in Early Post-Renal Transplant Period: A Meta-Analysis of Randomized Controlled Trials

**DOI:** 10.1371/journal.pone.0108106

**Published:** 2014-09-29

**Authors:** Zijie Wang, Zhijian Han, Jun Tao, Pei Lu, Xuzhong Liu, Jun Wang, Bian Wu, Zhengkai Huang, Changjun Yin, Ruoyun Tan, Min Gu

**Affiliations:** Department of urology, the first affiliated hospital of Nanjing Medical University, Nanjing, China; Ohio State University, United States of America

## Abstract

**Introduction:**

The overall effect of pamidronate on bone mass density (BMD) in the early renal transplant period varies considerably among studies. The effects of pamidronate on graft function have not been determined.

**Materials and Methods:**

A comprehensive search was conducted in PubMed, the Cochrane Central Register of Controlled Trials (CENTRAL) and Embase independently by two authors. Randomized controlled trials of pamidronate evaluating bone loss in the first year of renal transplantation were included. Methods reported in the “Cochrane Handbook for Systematic Reviews of Interventions 5.0.2” were used to evaluate changes of lumbar spine and femoral neck BMD, and serum creatinine, calcium and intact parathyroid hormone (iPTH) levels. Fixed or random effect models were used as appropriate.

**Results:**

Six randomized trials evaluating 281 patients were identified. One hundred forty-four were treated with pamidronate and 137 were control patients. Administration of pamidronate was associated with significant reduction of bone loss in the lumbar spine, compared to the control group (standardized mean difference (SMD)  = 24.62 [16.25, 32.99]). There was no difference between the pamidronate treated and control femoral neck BMD (SMD  = 3.53 [−1.84, 8.90]). A significant increase in the serum creatinine level of the intervention group was seen, compared to the control group. The serum calcium and iPTH of the pamidronate and control groups were not different after 1 year (serum creatinine: SMD  = −3.101 [−5.33, −0.89]; serum calcium: SMD  = 2.18 [−0.8, 5.16]; serum iPTH: SMD  = 0.06 [−0.19, 0.31]). Heterogeneity was low for serum calcium and iPTH and high for serum creatinine.

**Conclusions:**

This meta-analysis demonstrated the beneficial clinical efficacy of pamidronate on BMD with no association with any alteration in graft function during the first year of renal transplantation. Significant heterogeneity precludes the conclusion of the relationship between serum creatinine and pamidronate.

## Introduction

Kidney transplantation is an established treatment option for end-stage renal disease (ESRD) [1]. Bone mass density (BMD) loss induced by pre-transplantation bone disease, drug treatments for immunosuppression, secondary hyperparathyroidism and adynamic bone disease are major risk factors for complications such as infection and transplant rejection [2]. Smerud [3] reported that 0.5% of total femur and 1.9% of ultradistal radius BMD was lost during the first 12 months after a successful kidney transplant.

Pamidronate, a bisphosphonate (BP), is effective in preventing and treating post-transplant renal osteodystrophy. Pamidronate significantly reduces the rate of bone reabsorption and turnover and increases BMD. It maintains or improves the structural and material properties of bone and reduces the risk of fractures [4], [5]. Studies comparing pamidronate with traditional medicines, such as vitamin D and calcium, demonstrated pamidronate's effectiveness in protecting against early post-transplant bone loss [6], [7]. However, overall efficacy of pamidronate on bone loss during the early period of transplantation varies considerably across studies [6]–[9]. The safety of pamidronate on graft function in post-transplant recipients is not completely clear, although Lee S [10] has reported that pamidronate could attenuate post-renal transplant bone loss without leading to renal dysfunction. There are no previous meta-analyses of this topic.

## Methods

### Literature Search

A comprehensive search was conducted in PubMed, the Cochrane Central Register of Controlled Trials (CENTRAL) and Embase (updated on December 15th 2013) by two independent authors (Wang and Han). The Mesh search heading terms included “renal transplantation” or “kidney transplantation” combined with “pamidronate”. The reference lists of all studies included in the meta-analysis and abstracts of the Annual Meeting of the American Society of Nephrology, the International Transplant Society and the European Dialysis and Transplantation Association were also reviewed.

### Study Selection

The inclusion criteria were: (1) a randomized controlled trial (RCT) which investigated the use of pamidronate in renal transplant recipients with a control group receiving no treatment or placebo, alone or in combination with calcium and/or vitamin D in both groups; (2) a homogenous group of *de novo* adult renal transplant recipients; (3) at least one outcome of interest for our study. Two authors independently assessed the inclusion criteria and selected trials for final analysis. Disagreements were resolved after discussion.

### Study Quality

The quality of the eligible trials was assessed using the Jadad guidelines. Three specific domains, including random allocation, double-blinding and description of withdrawals and dropouts, were considered as the quality items [11]. A score of 0 to 5 was assigned to each study, 0 being the lowest and 5 being the best quality.

### Data Extraction

Changes in the BMD of the lumbar spine and femoral neck, and serum creatinine, calcium and intact parathyroid hormone (iPTH) levels from all eligible studies were extracted. Data extracted from previously published studies included study design, the size of the intervention and control groups, mean age of both groups, intervention protocol, dosage of pamidronate, immunosuppressive drug protocol, duration of the trial, and BMD at baseline and after follow-up. The standard deviation (SD) of the BMD of the lumbar spine and femoral neck of two studies and the mean and SD value of serum creatinine, calcium and iPTH of all eligible trials were estimated using a statistical method based on the Cochrane handbook [12]. Attempts were made to obtain missing data from the first or corresponding author of such studies.

### Statistical Analysis

BMD of the lumbar spine and femoral neck, and changes of serum creatinine, calcium and iPTH levels, were calculated separately from baseline to last follow-up in both groups. Data were analyzed using the methods of the “Cochrane Handbook for Systematic Reviews of Interventions 5.0.2”. *I^2^* was calculated to estimate heterogeneity among trials. *I^2^* was calculated as 100%* (Q-df)/Q, where Q was the Cochran's heterogeneity statistic and df was the number of degrees of freedom. A fixed-effect model set at low statistical inconsistency (*I^2^*<25%) was used. If *I^2^* was greater than 25%, a random-effects model was used [12], [13]. The average differences of each included trial were expressed as the standardized mean difference (SMD) and 95% confidence interval (CI). Forest plots were used to present overall results. STATA (release 12.0, College Station, TX) was used to complete all meta-analyses.

## Results

### Literature Search

**Figure 1:**
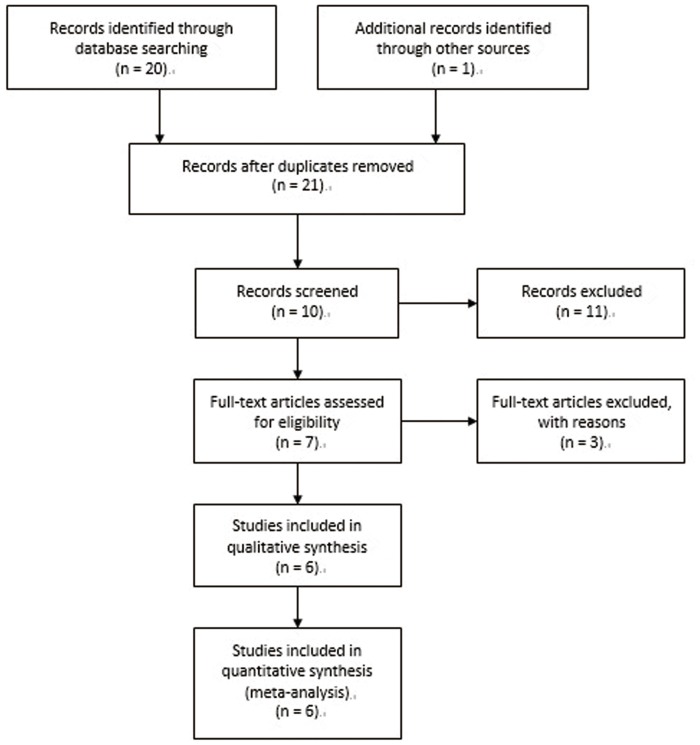
Flow diagram. Flow chart of trial selection.

Using the key words mentioned above, 21 citations were identified. Ten of these were selected for full-text review and 11 citations were excluded as they did not meet the inclusion criteria based on their titles or abstracts. Three of the 10 were excluded after full-text review, because they were case reports, did not have a full text, or were follow-up publications. One additional trial was excluded after attempting to contact the author due to the incompleteness of the available BMD values. Six RCTs with 281 participants were included in our meta-analysis [7]–[9], [14], [15] (Figure 1).

### Included trials

The characteristics and quality of the trails included are shown in Table 1. All trials administered intravenous pamidronate to the intervention group and oral calcium to both groups. In addition, oral cholecalciferol (800 IU/d) was supplied in one study for 12 months in both groups [8]. Another study added with Vitamin D_3_ [14]. Participants in two other studies received calcitriol in both the pamidronate group and the control group.

**Table 1:** Description of the 6 trials included in the meta-analysis.

Author/year/area	Sample size (PAMI/CON)	PAM group age (mean ±SD)	CON group age (mean ±SD)	Intervention	PAM administration (months after transplantation)	Control	Follow-up (months)	Immunosuppression	BMD measurements (months)	Quality score
**Nam JH/2000/South Korea(4)**	35(15/20)	44	44	PAM + calcium	30 mg i.v. (0, 1, 2, 3, 4, 5, 6 months)	Calcium	6	Not mentioned	0, 6	1
**Torregrosa JV/2011/Spain(7)**	29(19/10)	53.99±13.79	56.53±15.48	PAM + calcium + cholecalciferol	30 mg i.v. (7 d, 10 d, 3 months)	Placebo + calcium + cholecalciferol	12	Steroids + cyclosporine + MMF	0, 6, 12	4
**Fan SL/2000/UK(8)**	25(13/12)	53	50	PAM + calcium + Vit D	0.5 mg/kg i.v. (0, 1 month)	Placebo + calcium + Vit D	12	Steroids + AZA + cyclosporine	0, 3, 12	2
**Coco M/2003/US(9)**	59(31/28)	43.8±2.3	44.3±2.3	PAM + calcium + calcitriol	60 mg i.v. (0); 30 mg i.v. (1, 2, 3, 6 months)	Calcium + calcitriol	12	Steroids + cyclosporine/FK506	0, 6, 12	3
**Omidvar B/2011/Iran(10)**	40(20/20)	38.3	37.2	PAM+ calcium + calcitriol	90 mg i.v. (0, 1, 2, 3 months)	Alendronate + calcium + calcitriol	6	Steroids + cyclosporine + MMF	0, 6	2
**Walsh SB/2009/UK(4)**	93(46/47)	46.1±12.77	46.1±12.93	PAM + calcium + Vit D + cholecalciferol	1 mg/kg i.v. at 0, 1, 4, 8, 12 months	Calcium + Vit D + cholecalciferol	12	Steroids + cyclosporine	0, 3, 6, 12, 24	3

Abbreviation: PAM: pamidronate; CON: control; BMD: bone mineral density; SD: standard Deviation; Vit D: vitamin D; AZA: azathioprine; MMF: mycophenolate mofetil; FK506: tacrolimus.

Pamidronate was administered at doses ranging from 30 mg to 90 mg per intravenous injection. Two studies administered 0.5 mg/kg and 1.0 mg/kg pamidronate, respectively. Coco M [15] reported the lowest pamidronate treatment dose (60 mg) and Omidvar B [9] the highest.

BMD was determined using dual energy X-ray absorptiometry (DEXA) in six trials. Lumbar spine BMD change was reported in five studies and BMD changes of the femoral neck were reported in four studies [15]. Serum creatinine, calcium and iPTH levels were reported in four trials. All eligible studies performed administration of pamidronate and placebo agents preemptively as no osteoporosis was present in enrolled patients.

The quality of trials involved was assessed by two independent authors using Jadad guidelines [11] for estimating the risk of bias. One study [8] received a score of 4 for double-blinding without detailed explanations. Other reports [6], [7], [9], [14], [15] were not scored above 3 due to the lack of double-blinding.

### Quantitative Data Analysis

We selected the later data point if BMD was evaluated at both 6 months and 12 months. Changes of BMD in the lumbar spine, femoral neck and serum creatinine, calcium, and iPTH levels were analyzed in our meta-analysis.

**Figure 2:**
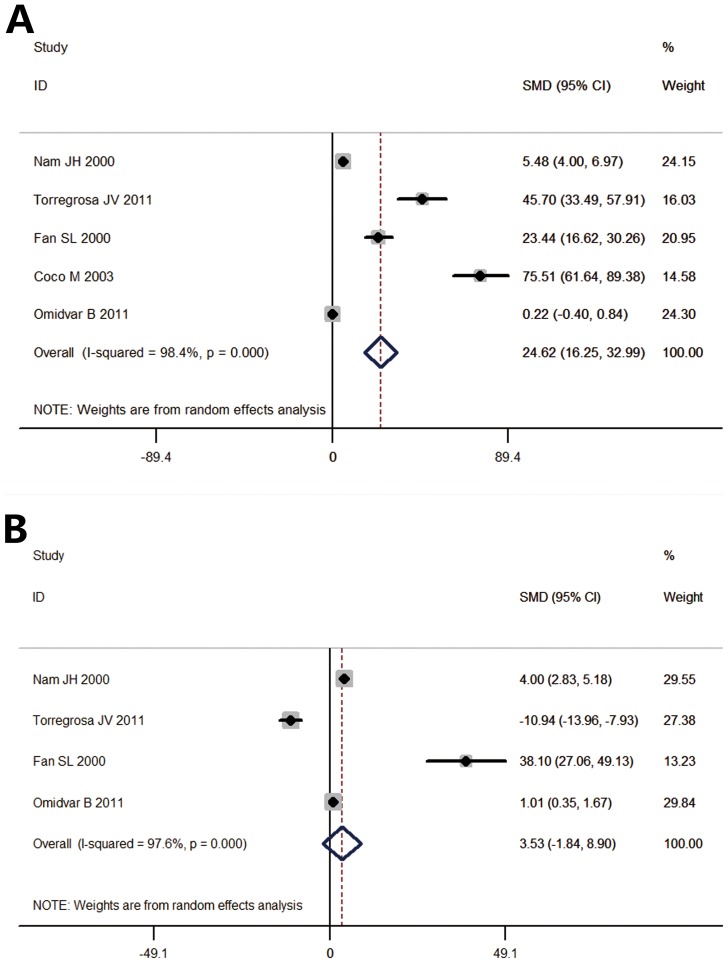
Forest plot of lumbar spine and femoral neck BMD change. (A). The administration of pamidronate was associated with significant benefit to the intervention group, compared to the control group. (SMD  = 24.62 [16.25, 32.99], *p* for effect <0.001, *p* for heterogeneity <0.001, *I^2^* = 98.4%). Five studies with 188 patients were analyzed. (B). No significant difference was found in the BMD of the intervention and control groups (SMD  = 3.53 [−1.84, 8.90], *p* for effect  = 0.198, *p* for heterogeneity <0.001, *I^2^* = 97.6%). Four studies with 129 patients were analyzed. BMD: bone mineral density; SMD: standardized mean difference.

The pamidronate treated group had significantly less decline in lumbar spine BMD than the control group (Figure 2) (SMD  = 24.62 [16.25, 32.99], *p* for effect <0.001, *p* for heterogeneity <0.001, *I^2^* = 98.4%). Five studies with 188 patients were evaluated. A random effects model was used to evaluate the femoral neck (Figure 2). The pamidronate treated group and control group had similar declines in femoral neck BMD (SMD  = 3.53 [−1.84, 8.90], *p* for effect  = 0.198, *p* for heterogeneity <0.001, *I^2^* = 97.6%). Four studies with 129 patients were evaluated.

**Figure 3:**
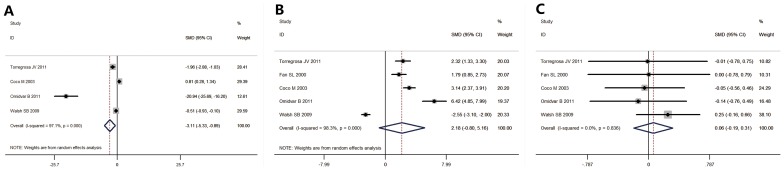
Forest plot of the change in serum creatinine, calcium and iPTH. (A). There was a significant increase in the serum creatinine of the intervention group (SMD  = −3.101 [−5.33, −0.89], *p* for effect  = 0.006, *p* for heterogeneity <0.001, *I^2^* = 97.1%). Four studies with 221 patients were analyzed.(B). No significant difference was detected in the serum calcium of the intervention and control groups (serum calcium: SMD  = 2.18 [−0.8, 5.16], *p* for effect  = 0.151, *p* for heterogeneity <0.001, *I^2^* = 98.3%). Five studies with 246 patients were analyzed. (C). There was no difference in the serum iPTH of the intervention and control groups at 1-year follow-up (SMD  = 0.06 [−0.19, 0.31], *p* for effect  = 0.646, *p* for heterogeneity  = 0.836, *I^2^* = 0.00%). Five studies with 246 patients were analyzed. iPTH: intact parathyroid hormone. SMD: standardized mean difference.

A significant increase in the serum creatinine (Figure 3) was found in the intervention group, and control group (SMD  = −3.101 [−5.33, −0.89], *p* for effect  = 0.006, *p* for heterogeneity <0.001, *I^2^* = 97.1%). Four studies with 221 patients were evaluated. There was no difference in the serum calcium (Figure 3) levels of the two groups (SMD  = 2.18 [−0.8, 5.16], *p* for effect  = 0.151, *p* for heterogeneity <0.001, *I^2^* = 98.3%). Five studies with 246 patients were evaluated. *I^2^* from the I-squared test was 0.00% for iPTH, so a fixed effect model was used. The pamidronate and control groups had similar serum iPTH levels (Figure 3) at 1 year (SMD  = 0.06 [−0.19, 0.31], *p* for effect  = 0.646, *p* for heterogeneity  = 0.836, *I^2^* = 0.00%). Five studies with 246 patients were evaluated.

Sensitivity analysis was performed. BMD of the lumbar spine and femoral neck and serum calcium findings were not altered by omitting any single trial. Significant heterogeneity was identified in the serum creatinine analysis. No study characteristic was related to the lack of homogeneity in the relationship between serum creatinine and pamidronate.

### Bone fractures and adverse effects

Coco M [15] reported three new vertebral fractures at 12 months, one in the pamidronate group and two in the control group. Torregrosa [8] reported one peripheral fracture at 6 months in the pamidronate group. No other new fractures were reported in the other four studies.

Walsh [6] reported 5 episodes of transient hypocalcemia (8.6%) in pamidronate group which could be considered as pamidronate-related adverse events. No withdrawal of pamidronate administration was reported during experiment due to adverse effects.

### Graft function and immunosuppression

There was no acute rejection reported due to the administration of pamidronate in any of the trials included. Torregrosa JV [8] observed seven rejections, four in the pamidronate group and three in control group, during follow-up. This constituted 18% of the renal transplant cases. There was no difference in the incidence of acute rejection of the two groups.

Two-drug or three-drug immunosuppressive regimens, including steroids and calcineurin inhibitors such as cyclosporine and FK506 (tacrolimus), were used in five trials. No induction phase was reported in the trial reported by Nam [7]. There was no significant difference in the doses of immunosuppressive drugs administered to the two groups.

## Discussion

Nitrogen-containing BPs such as Alendronate and Pamidronate are taken up preferentially by the skeleton and suppress bone resorption. They are widely used in the treatment of Paget's disease of bone, metastatic and osteolytic bone disease, hypercalcemia of malignancy, and glucocorticoid-induced osteoporosis [4], [16]. Recipients of kidney transplantation may experience rapid bone loss during the first 12–18 months and may continue to undergo persistent bone loss for many years, which could lead to post-transplant osteoporosis [17], [18].Practice guidelines for kidney transplantation recommend vitamin D and calcium supplements in the absence of contraindications and BPs for the prevention of bone loss in the early post-transplant period [19], [20]. All six studies performed pamidroante administration after kidney transplantation preemptively, which could be considered as prophylaxis for post-transplant rapid bone loss and osteoporosis. Outcomes of our meta-analysis are consistent with the mechanism of action of BPs mentioned above and the recommendation of these guidelines.

Our meta-analysis confirmed that pamidronate reduced bone loss of the lumbar spine and not the femoral neck. Bone cells have heterogeneous responses to pamidronate according to whether the bone is cancellous or cortical. Cortical osteoclasts seem to be unaffected by the use of BPs [21], possibly accounting for the different BMD outcomes in the lumbar spine and femoral neck. Boyce [22] reported that human parathyroid hormone (1–34) [hPTH (1–34)] plus risedronate was superior to hPTH (1–34) plus 1, 25(OH) _2_ D _3_ in preventing osteoporosis of the cortical envelope. The action of iPTH and pamidronate requires a long time to become apparent. Thus, longer follow-up may be needed to demonstrate the protective efficacy of pamidronate on the femoral neck.

Administration of pamidronate was not associated with renal toxicity during the first year of kidney transplantation since there was no significant difference in the relationship between serum calcium, iPTH and the administration of pamidronate. In contrast, some other BPs including alendronate, have been shown to have some detrimental effect on renal function [23]–[25].However, a slight increase in serum creatinine level with a high degree of heterogeneity was seen. No study characteristic was found to be related to the lack of homogeneity in the relationship between serum creatinine and pamidronate. Different treatment criteria, different dosages used and different durations of treatment in 4 of the trials may have contributed to this heterogeneity. In addition to the poor quality of included articles, the pooled data of serum creatinie must be interpreted with caution and the relationship between serum creatine and pamidronate remained to be determined.

The optimal drug to prevent BMD should provide predictable effects, be easy to administer with no adverse events, and have no withdrawal effects at the end of infusion [26]. Pamidronate is well-tolerated, long-lasting and needs minimal additional monitoring during treatment. In contrast, alendronate is associated with gastrointestinal complications after sudden withdrawal of the medication. None of the 6 trials reported serious adverse events from pamidronate. Pamidronate administration is sometimes associated with fever and flu-like symptoms at the start of treatment, the so-called “post-dose” symptoms. These effects are transient and occur predominantly after the first intravenous administration of pamidronate, probably due to the release of pro-inflammatory cytokines [16], [27], [28]. Moreover, jaw osteonecrosis and atypical femoral fractures were also reported in few trials with administration of pamidronate in the general population, which remained to be observed in transplant recipients[29], [30].Torregrosa [8] used low doses of pamidronate (30 mg i.v., on days 7 and 10, and 3 months after transplant) to reduce bone turnover, a simple regimen to administer regimen.

Some new bone turnover markers, including iPTH, osteocalcin (OC), procollagen type I N propeptide (PINP), serum C -terminal cross-linking telopeptide of type I collagen (sCTX) and bone-specific alkaline phosphatase (BSAP) have been used to monitor bone remodeling and bone reabsorption. Torregrosa [8] analyzed the performance of PINP and CTX in administration of pamidronate. The bone remodeling markers of PINP and CTX fall initially during the first 3 months in pamidronate group and control group, and distinct outcomes, recovering starting in control group and continuous slight decrease in pamidronate group, was found during 12 months, although differences between two groups were not significant. The experience with all these, except for iPTH, is limited. We did not observe any significant differences in the iPTH levels of the pamidronate and control groups. Biomarkers specific to bone turnover and remodeling [31], such as CTX in urine and serum, PINP and OC, might be more sensitive indicators of bone metabolism. Their efficacy remains to be validated in clinical studies.

Our meta-analysis had some limitations. Roschger [32] showed that cancellous and cortical bone are both strengthened by the BPs alendronate and pamidronate. This was confirmed by a four-year study of bone loss with pamidronate [33]. In contrast, we did not see a positive outcome in the femoral neck. This may be due to the relatively short, one-year follow-up time. Longer studies are needed to better examine this treatment effect. The small number and low quality of some ariticles introduced a high risk of bias. A large number of multicenter randomized controlled trials with long term follow-up would better evaluate the actions of pamidronate.

In conclusion, our meta-analysis suggests that pamidronate, which is simple to administer and well tolerated without any serious adverse effects, is beneficial to bone loss and is not correlated with renal toxicity in the first year after renal transplantation. Further clinical studies are needed to confirm our conclusions. The best way to monitor these patients' bone turnover is yet to be determined.

## Supporting Information

Checklist S1 (DOCX)
